# A quantitative modeling framework to understand the physiology of the hypothalamic-pituitary-adrenal axis and interaction with cortisol replacement therapy

**DOI:** 10.1007/s10928-024-09934-7

**Published:** 2024-07-08

**Authors:** Davide Bindellini, Robin Michelet, Linda B. S. Aulin, Johanna Melin, Uta Neumann, Oliver Blankenstein, Wilhelm Huisinga, Martin J. Whitaker, Richard Ross, Charlotte Kloft

**Affiliations:** 1 Dept. of Clinical Pharmacy and Biochemistry, Institute of Pharmacy, Freie Universitaet Berlin, Berlin, Germany; 2 Graduate Research Training program PharMetrX, Berlin, Germany; 3 Charité-Universitätsmedizin Berlin, Berlin, Germany; 4 Labor Berlin, Charité Vivantes GmbH, Berlin, Germany; 5 Institute of Mathematics, University of Potsdam, Potsdam, Germany; 6 University of Sheffield, Sheffield, UK

**Keywords:** Congenital adrenal hyperplasia, Circadian rhythm, Cortisol, ACTH, Modeling, Cortisol replacement therapy

## Abstract

Congenital adrenal hyperplasia (CAH) is characterized by impaired adrenal cortisol production. Hydrocortisone (synthetic cortisol) is the drug-of-choice for cortisol replacement therapy, aiming to mimic physiological cortisol circadian rhythm. The hypothalamic-pituitary-adrenal (HPA) axis controls cortisol production through the pituitary adrenocorticotropic hormone (ACTH) and feedback mechanisms. The aim of this study was to quantify key mechanisms involved in the HPA axis activity regulation and their interaction with hydrocortisone therapy. Data from 30 healthy volunteers was leveraged: Endogenous ACTH and cortisol concentrations without any intervention as well as cortisol concentrations measured after dexamethasone suppression and single dose administration of (i) 0.5–10 mg hydrocortisone as granules, (ii) 20 mg hydrocortisone as granules and intravenous bolus. A stepwise model development workflow was used: A newly developed model for endogenous ACTH and cortisol was merged with a refined hydrocortisone pharmacokinetic model. The joint model was used to simulate ACTH and cortisol trajectories in CAH patients with varying degrees of enzyme deficiency, with or without hydrocortisone administration, and healthy individuals. Time-dependent ACTH-driven endogenous cortisol production and cortisol-mediated feedback inhibition of ACTH secretion processes were quantified and implemented in the model. Comparison of simulated ACTH and cortisol trajectories between CAH patients and healthy individuals showed the importance of administering hydrocortisone before morning ACTH secretion peak time to suppress ACTH overproduction observed in untreated CAH patients. The developed framework allowed to gain insights on the physiological mechanisms of the HPA axis regulation, its perturbations in CAH and interaction with hydrocortisone administration, paving the way towards cortisol replacement therapy optimization.

**Graphical Abstract:**

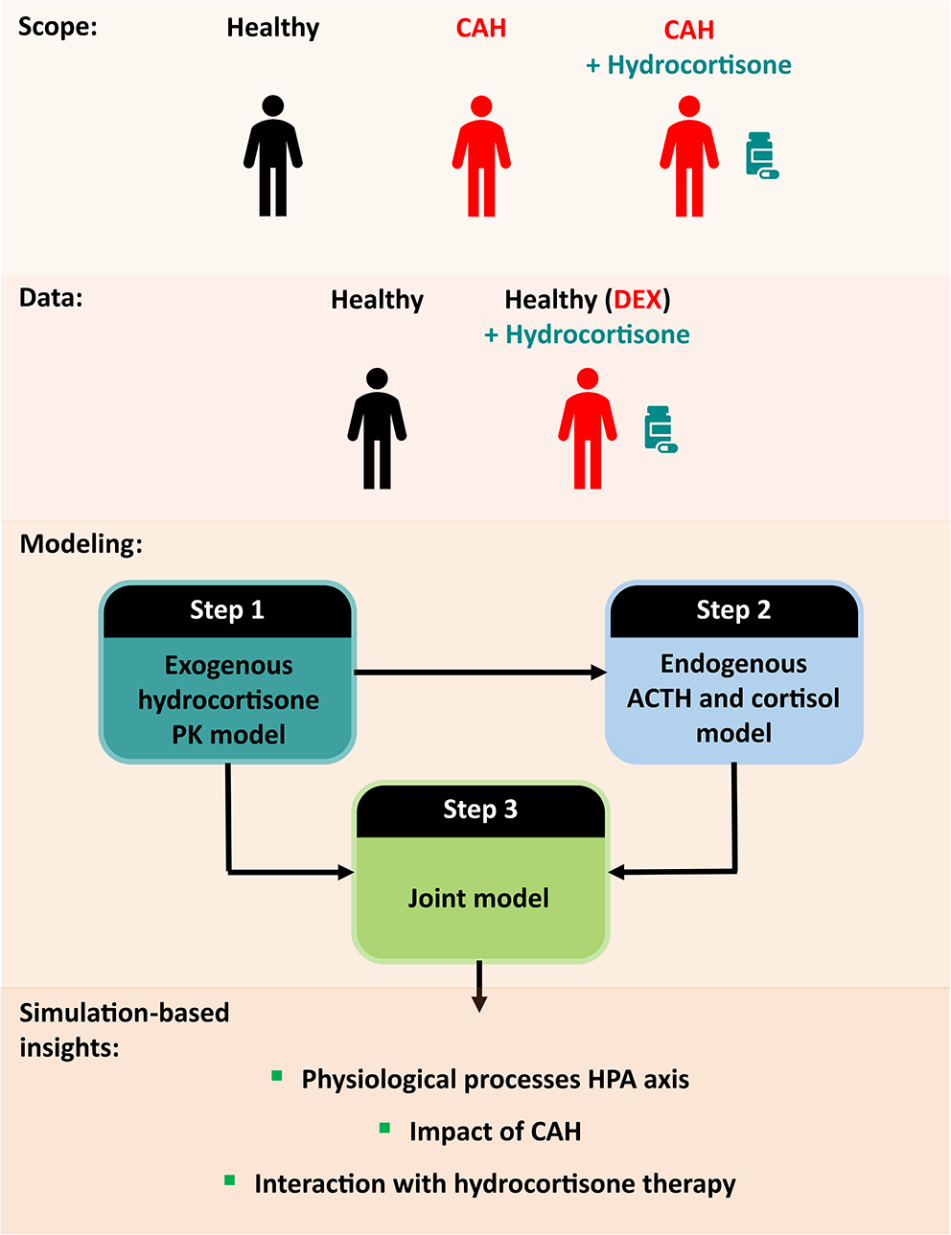

**Supplementary Information:**

The online version contains supplementary material available at 10.1007/s10928-024-09934-7.

## Introduction

Congenital adrenal hyperplasia (CAH) is the most common inherited endocrine disorder of the adrenal gland resulting from mutations in the steroid enzyme 21-hydroxylase gene, leading to a deficiency in the adrenal hormone cortisol [1]. The severity of cortisol deficiency depends on the degree of enzymatic deficiency [2, 3]: In severe cases, there is no remaining enzymatic activity, while for intermediate and mild CAH cases the remaining enzymatic activity is 1-2% and 20-50% respectively [4–6]. Ultimately, for untreated severe cases, cortisol deficiency results in death through adrenal crisis [2, 3].

Cortisol production follows a circadian rhythm driven by the central clock in the hypothalamus. This process is mediated by corticotropin-releasing hormone (CRH), which induces the secretion of adrenocorticotropic hormone (ACTH) from the pituitary gland. Subsequently, ACTH stimulates the adrenal gland to produce cortisol. This endocrine pathway is often referred to as the hypothalamic-pituitary-adrenal (HPA) axis [7–10]. In the healthy state, the homeostasis of the cortisol circadian rhythm is maintained by a cortisol-driven feedback inhibition on CRH and ACTH secretion [7, 10, 11].

**Fig. 1:**
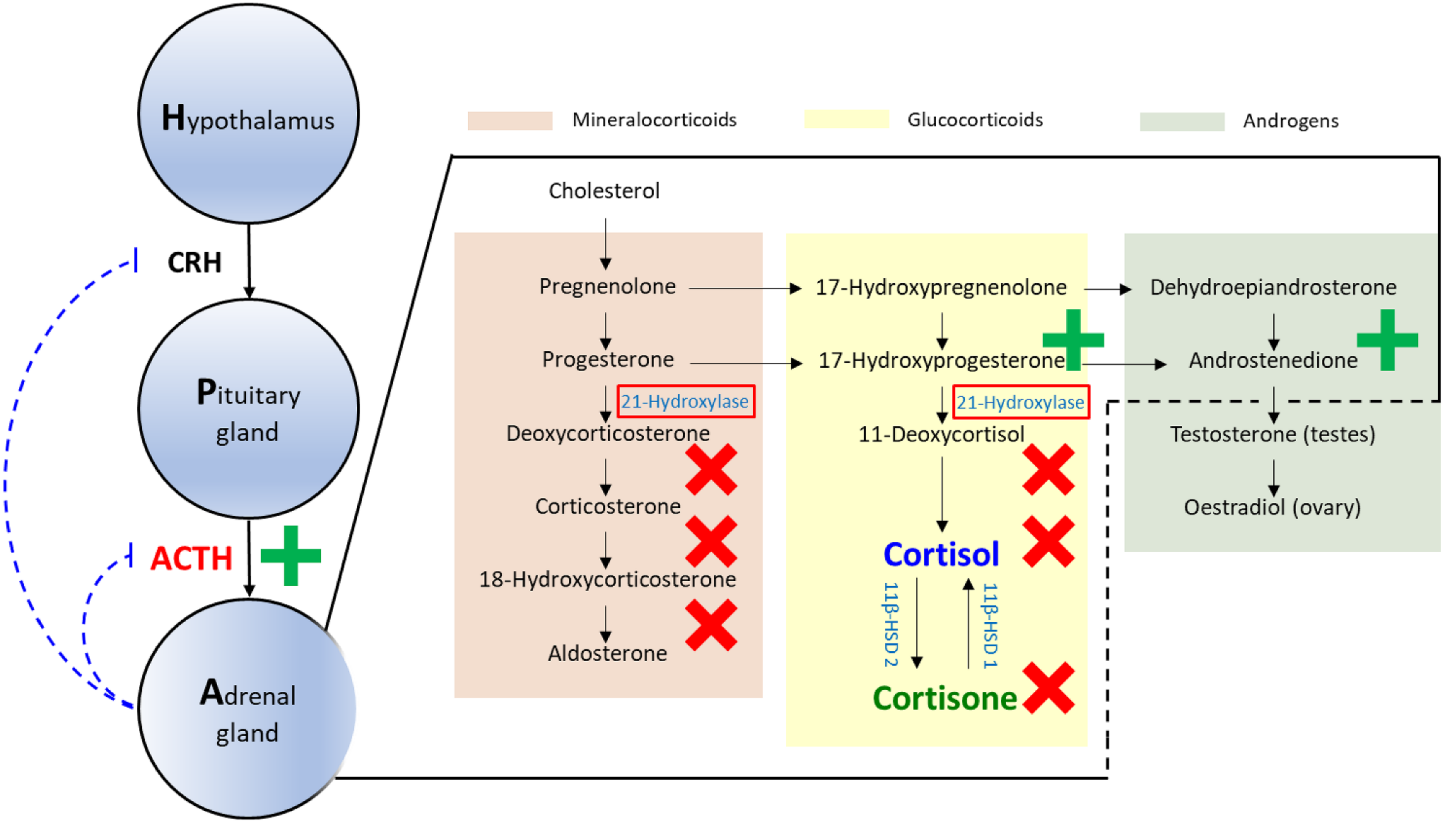
Hypothalamic-pituitary-adrenal axis and cortisol related pathways (red, green and yellow areas: Mineralocorticoid, androgens and glucocorticoid pathways, respectively) and their alterations in congenital adrenal hyperplasia with red box: deficient enzyme, red crosses: species with impaired production, green plus: overproduced species. ACTH: Adrenocorticotropic hormone, CRH: Corticotropic releasing hormone, 11β-HSD 1: 11β dehydrogenase type 1, 11β-HSD 2: 11β dehydrogenase type 2

In CAH patients, the lack of cortisol-driven feedback inhibition results in excess secretion of ACTH and dysregulation of adrenal hormone production (Fig. 1). Moreover in CAH, the excess ACTH drives the production of adrenal cortisol precursors and results in overproduction of adrenal androgens [2, 3]. The overproduction of androgens causes virilization of the female infant, premature pseudo puberty and short stature in children, virilization in women and infertility in both men and women [2, 3]. Additionally, in CAH patients, aldosterone production is also deficient because of the mutations in the steroid enzyme 21-hydroxylase gene (Fig. 1): In severe cases, this can result in fatal electrolyte imbalances.

Congenital adrenal hyperplasia patients require lifelong cortisol replacement therapy, with hydrocortisone (synthetic cortisol) being the preferred drug for cortisol replacement [3, 12]. Current cortisol replacement treatment with immediate-release hydrocortisone in CAH is challenging as it fails to mimic endogenous cortisol circadian rhythm [13] and ACTH excess still occurs in treated patients, particularly in the early morning. To optimize cortisol replacement therapy, and the design of hydrocortisone dosing regimens and formulations, it is essential to quantitatively characterize the physiology of the HPA axis and how its components interact with hydrocortisone therapy.

To date, many efforts have been made to characterize hydrocortisone pharmacokinetics (PK) in adults and children [14–21]. Characterizations of the impact of CAH on the physiology of the HPA axis have primarily been derived from animal models [22, 23]. A quantitative framework incorporating current knowledge on the HPA-cortisol pathway in healthy adults, its alterations in CAH, and interaction with hydrocortisone therapy is still missing. To address this knowledge gap, we postulate that nonlinear mixed-effects (NLME) modeling can be applied as an approach to incorporate different sources of data and variability into one coherent framework.

The aim of this study was to gain quantitative insights on the HPA-cortisol pathway in the healthy state, in CAH, and its perturbation by hydrocortisone administration. Thus, a quantitative modeling framework incorporating physiological processes of the HPA axis and hydrocortisone PK was developed. Ultimately, to evaluate the impact of CAH and cortisol replacement therapy on the HPA-cortisol production pathway, the model should be leveraged to perform simulations of CAH patients with varying remaining enzymatic activity, with and without hydrocortisone administration, and healthy individuals. In this study, such simulations were performed as proof of concept.

## Methods

### ACTH and cortisol clinical trial database

**Fig. 2:**
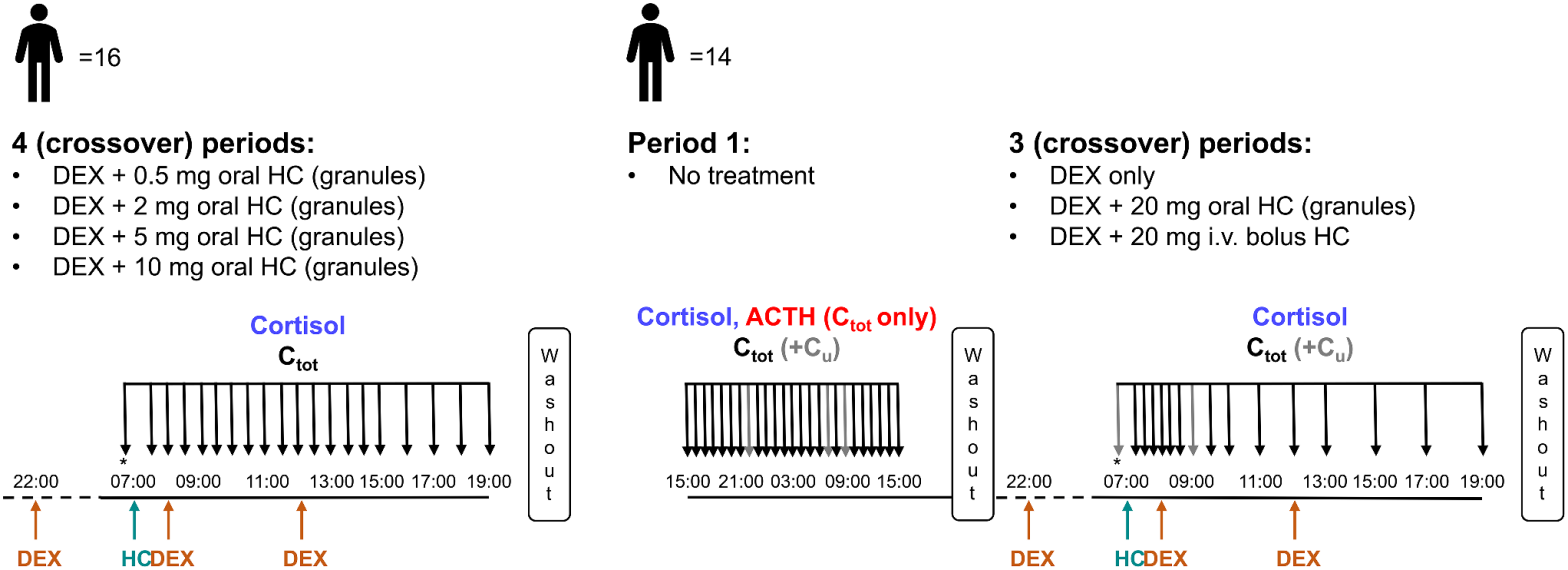
Schematic representation of the two clinical trial designs. Orange and cyan arrows: DEX and HC administration time points, respectively; black and gray arrows: Sampling time points (C_tot_: total cortisol and ACTH (study 2, period 1)) and (C_tot_+C_u_: total and unbound cortisol), respectively. *ACTH measured in pre-dose samples. ACTH: Adrenocorticotropic hormone, C_tot_: Total concentration, C_u_: Unbound concentration, DEX: Dexamethasone, HC: Hydrocortisone

Data from two cross-over phase I clinical trials (NCT02777268, NCT01960530) in healthy male adults approved by the South East Wales Research Ethics committee were used in this work [24, 25]. The first trial (*N* = 16) [24] consisted of four periods in a randomized order. The participants were given multiple dexamethasone (DEX) doses to suppress endogenous cortisol production, in this way the effect of exogenous hydrocortisone could be investigated. Pre-hydrocortisone dosing ACTH and cortisol total concentrations were measured to ensure that DEX-mediated suppression was successful. Participants were administered a single dose of 0.5, 2, 5 and 10 mg hydrocortisone immediate-release granules at 07:00. Total cortisol concentrations were measured pre-dose and half hourly up to 8 h and hourly up to 12 h after hydrocortisone administration. A washout period of at least one week was realized between each trial period (Fig. 2).

The second trial (*N* = 14) [25] consisted of a first trial period in which neither DEX nor hydrocortisone were given to the participants and endogenous ACTH and endogenous total cortisol concentrations were measured in the healthy state every hour over 24 h (from 15:00 to 15:00). Furthermore, endogenous unbound cortisol concentrations were obtained for the samples collected at 22:00, 07:00 and 09:00. The participants were then given in a randomized order of three periods: multiple doses of DEX only, or multiple doses of DEX plus either a single dose of 20 mg hydrocortisone as immediate-release granules or as intravenous (i.v.) bolus. Total cortisol concentrations were measured pre-dose and at 0.25, 0.5, 0.75, 1, 1.25, 1.5, 2, 2.5, 3, 4, 5, 6, 8, 10 and 12 h after period start (07:00). Unbound cortisol concentrations were obtained from the samples collected pre-dose and 2 h post-dose/period start. A washout period of at least one week was realized between each trial period (Fig. 2). Further details regarding samples preparation and bioanalytical quantification methods were previously published [20, 26].

### Stepwise modeling workflow

Through the development of the endogenous ACTH cortisol model, cortisol PK parameters would not be identifiable, affecting the characterization and quantification of relevant processes of the HPA axis. Thus, under the assumption that cortisol and hydrocortisone follow identical PK behavior, a previously developed hydrocortisone PK model was used as a starting point for the modeling activities [20]. Empirical Bayes Estimates (EBEs) were extracted from this model to characterize endogenous cortisol PK in the participants from the second trial. However, since a misspecification in the absorption characterization for the 20 mg immediate-release granules trial period was observed, a refinement of the absorption model was necessary to obtain unbiased EBEs. Therefore, the modeling workflow was structured as follows:

Step 1: Refinement of the absorption characterization in the previously developed hydrocortisone PK model. All data from the first trial, as well as DEX only, DEX + hydrocortisone granules and DEX + hydrocortisone i.v. bolus from the second trial (Fig. 2) were leveraged.

Step 2: Development of a model for endogenous ACTH and endogenous cortisol (cortisol PK fixed using hydrocortisone PK EBEs obtained from Step 1). Data from the first period of the second trial (ACTH and cortisol endogenous concentrations) as well as from the DEX only trial period of the second trial (Fig. 2) were leveraged.

Step 3: Integration of endogenous ACTH and endogenous cortisol and hydrocortisone PK models in a joint model.

### Step 1: hydrocortisone PK model refinement

The previously developed hydrocortisone PK model was a two-compartment disposition model that included a saturable absorption process of hydrocortisone, hydrocortisone binding to corticosteroid binding globulin and albumin and theory-based allometric scaling for volume and flow parameters [20]: Only the absorption process was re-evaluated and refined in this work. Two alternative more complex absorption models were evaluated, referred to as ABS1 (“split-dose”) model and ABS2 (“estimating number of transit compartments”) model, respectively. In the ABS1 model, the dose was split into two depot compartments, one with first-order absorption and one with transit compartments for absorption: The use of 2, 3, 4 and 5 transit compartments was evaluated. The fraction of dose being absorbed from the first depot compartment was estimated (FA), while the remaining fraction was absorbed from the second depot compartment (1-FA). The ABS2 model was a more commonly used transit compartment absorption model with estimated number of transit compartments [27]. The models ABS1 and ABS2 were developed and their performance compared. For both models, interindividual variability (IIV) models on absorption parameters were evaluated. Additionally, for the individuals from the first trial, interoccasion variability (IOV) models on absorption parameters were evaluated. Lastly, dose-dependencies on absorption parameters in both ABS1 and ABS2 models were explored.

### Step 2: endogenous ACTH and cortisol model

To characterize ACTH time-dependent pulsatile secretion, surge functions were used in combination with a baseline secretion modeled by zero-order secretion plus first-order elimination. Surge functions (Eq. 1) are characterized by an amplitude (SA), width (SW), peak time (Pt) and an exponent (n) which dictates the shape of the resulting peak: The higher the exponent, the flatter the peak. The exponent n can only assume even positive values: 2, 4, 6 and 8 were evaluated. Pulsatile secretion models with two and three surge functions were evaluated.

1 $$S\left(t\right)=\frac{SA}{{\left(\raisebox{1ex}{$t-Pt$}\!\left/ \!\raisebox{-1ex}{$SW$}\right.\right)}^{n}+1}$$

Interindividual variability was evaluated on all ACTH-related parameters before including cortisol data. Lastly, additive, proportional and combined residual unexplained variability (RUV) models were tested.

Cortisol production rate was assumed to depend exclusively on ACTH concentrations. For the ACTH concentration to cortisol production rate relationship linear, log-linear, E_max_ and sigmoidal E_max_ models were evaluated. For the quantitative characteristics of the cortisol distribution, protein binding and elimination processes, hydrocortisone PK EBEs were extracted from the most appropriate model from Step 1 and used as individual cortisol PK parameters in the development of the endogenous model. Then, the implementation of a feedback inhibition from cortisol unbound concentrations onto ACTH pulsatile secretion was evaluated using hyperbolic and sigmoidal I_max_ models. The negative feedback was assumed to be able to fully suppress ACTH pulsatile secretion. Similarly, DEX administration was implemented by fully suppressing ACTH pulsatile secretion in the trial periods when it was given, with and without hydrocortisone. Then, IIV was evaluated on cortisol production-related parameters as well as ACTH suppression-related parameters. Additive, proportional and combined RUV models were tested.

To evaluate the effect of body weight and age on all parameters on which IIV was included, a covariate analysis was performed by stepwise covariate modeling (SCM) using significance levels of 0.05 for forward inclusion and 0.005 for backward deletion: Linear, power and exponential relationships between covariates and parameters were evaluated.

### Step 3: joint model parameters re-estimation

The developed endogenous ACTH and cortisol model from Step 2 and the refined hydrocortisone PK model from Step 1 were integrated into a single joint model. In this last step, all non-fixed parameters were re-estimated simultaneously.

### Model evaluation

Intermediate models from Step 1 and Step 2 were evaluated based on parameter plausibility and precision, goodness of fit (GOF) plots and, if nested, compared based on the difference in objective function value (dOFV): For the comparison of the best ABS1 and ABS2 models Akaike information criterion was used (AIC) instead of OFV, since the models were not nested. The predictive performance of key intermediate models and the selected models from each step was also evaluated by visual predictive check (VPC, *n* = 1000). Additionally, the parameters precision of the selected models from each step was evaluated using sampling importance resampling (SIR).

### Simulations: CAH patients and healthy individuals

To generate CAH patients with different extents of disease severity and to compare ACTH and cortisol concentrations in CAH and healthy state, the developed model from Step 3 was used to simulate (*n* = 1000) ACTH and cortisol concentration trajectories in 70 kg patients of different CAH severity and healthy individuals. CAH patients were simulated by assuming no (0% remaining enzymatic activity, severe CAH) or decreased (20% remaining enzymatic activity, mild CAH) endogenous cortisol production.

### Simulations: Interaction with hydrocortisone therapy

The reduction in ACTH overproduction was used as a metric to evaluate optimal hydrocortisone dosing time in CAH patients with no endogenous cortisol production (severe CAH patients). In particular, ACTH overproduction was compared in severe CAH patients without hydrocortisone, and when 10 mg immediate-release hydrocortisone granules were given at 05:00 or 07:00.

### Software

PsN (Perl Speaks NONMEM) v4.8.1 was used to access NONMEM v7.4.3 through Pirana v2.9.6 to perform modelling and simulation activities, while data management, data visualization and processing of modeling results were performed using R v4.2.1 with RStudio v2022.07.2 + 576.

## Results

### ACTH and cortisol clinical trial database

One participant from the second trial was excluded from the analysis as endogenous ACTH suppression was not sufficient (pre-dose ACTH concentration > 20 pg/mL compared to pre-dose ACTH concentration < 5 pg/mL typically observed in DEX-suppressed individuals [28]). A total of 1865 total cortisol concentrations from the intervention trial periods (DEX/hydrocortisone administration) as well as 325 total endogenous cortisol concentrations and 310 endogenous ACTH concentrations were available for analysis. The participants´ characteristics were comparable across the two clinical trials (Table 1): The total population included in this study (*n* = 29) was young to middle-aged (median (range)) (38.5 years (21.0–60.0)) and normal body weight (81.7 kg (64.7–102)).

**Table 1:** Study participants characteristics for covariate analysis

Covariates [unit]	Study 1 (*n* = 16)	Study 2 (*n* = 13)	Total (*n* = 29)
	Median (range)	Median (range)	Median (range)
Body weight [kg]	81.5 (64.7–96.0)	83.3 (66.6–102)	81.7 (64.7–102)
Age [years]	39.0 (21.0–59.0)	29.5 (22.0–60.0)	38.5 (21.0–60.0)

The following Step 1 and Step 2 sections provide an overview of the model structure and mathematical functions that best characterized the processes and the data, while parameters value and their interpretation will be provided under Step 3 section.

### Step 1: hydrocortisone PK model refinement

**Fig. 3:**
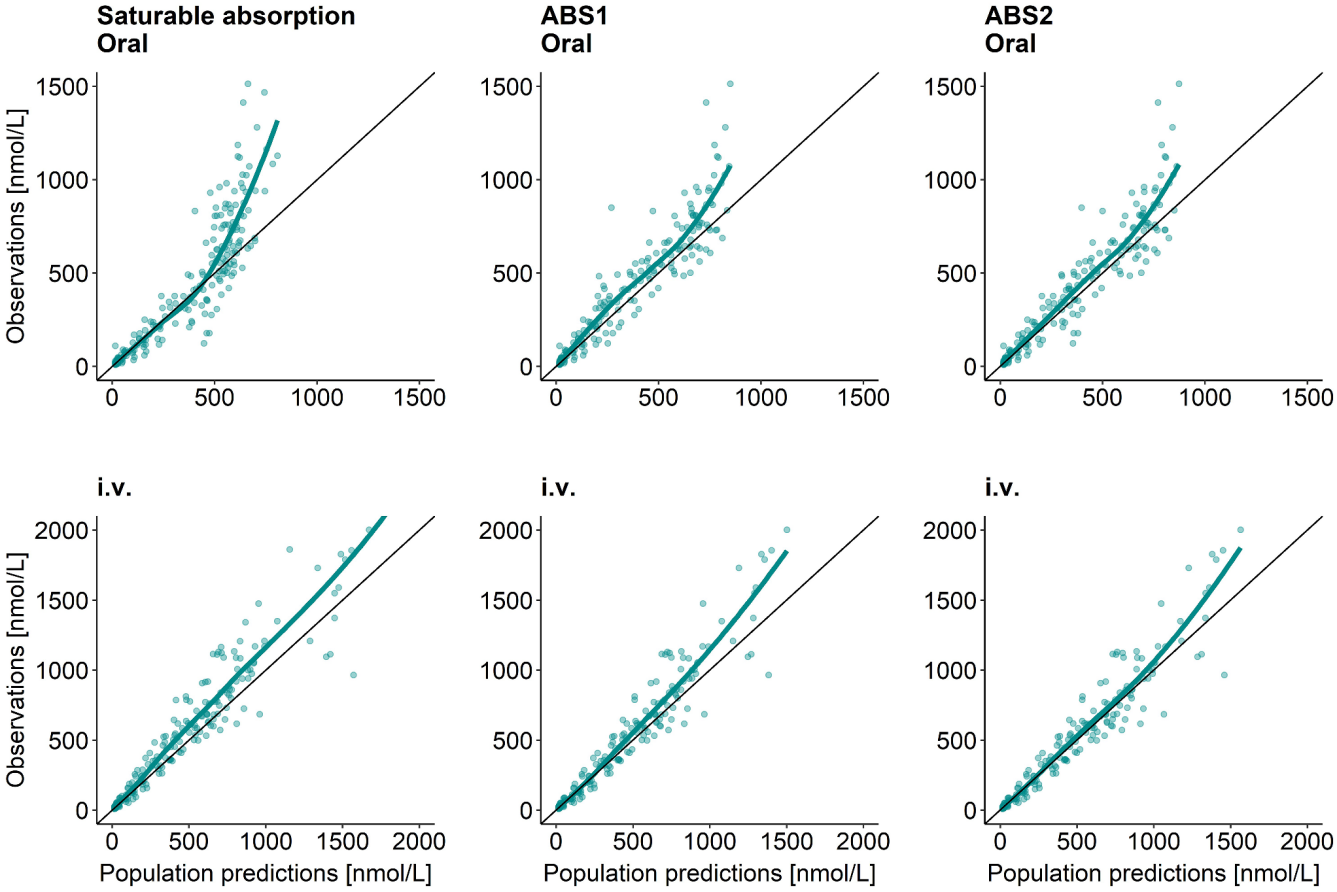
Goodness of fit plots (observations versus population predictions for different hydrocortisone absorption models: Left panels for the original saturable absorption model [20], middle panels for developed ABS1 (“split-dose”) model, right panels for developed ABS2 (“estimating number of transit compartments”) model. Top panels for 20 mg oral hydrocortisone administration and bottom panels for 20 mg intravenous (i.v.) bolus hydrocortisone administration

**Fig. 4:**
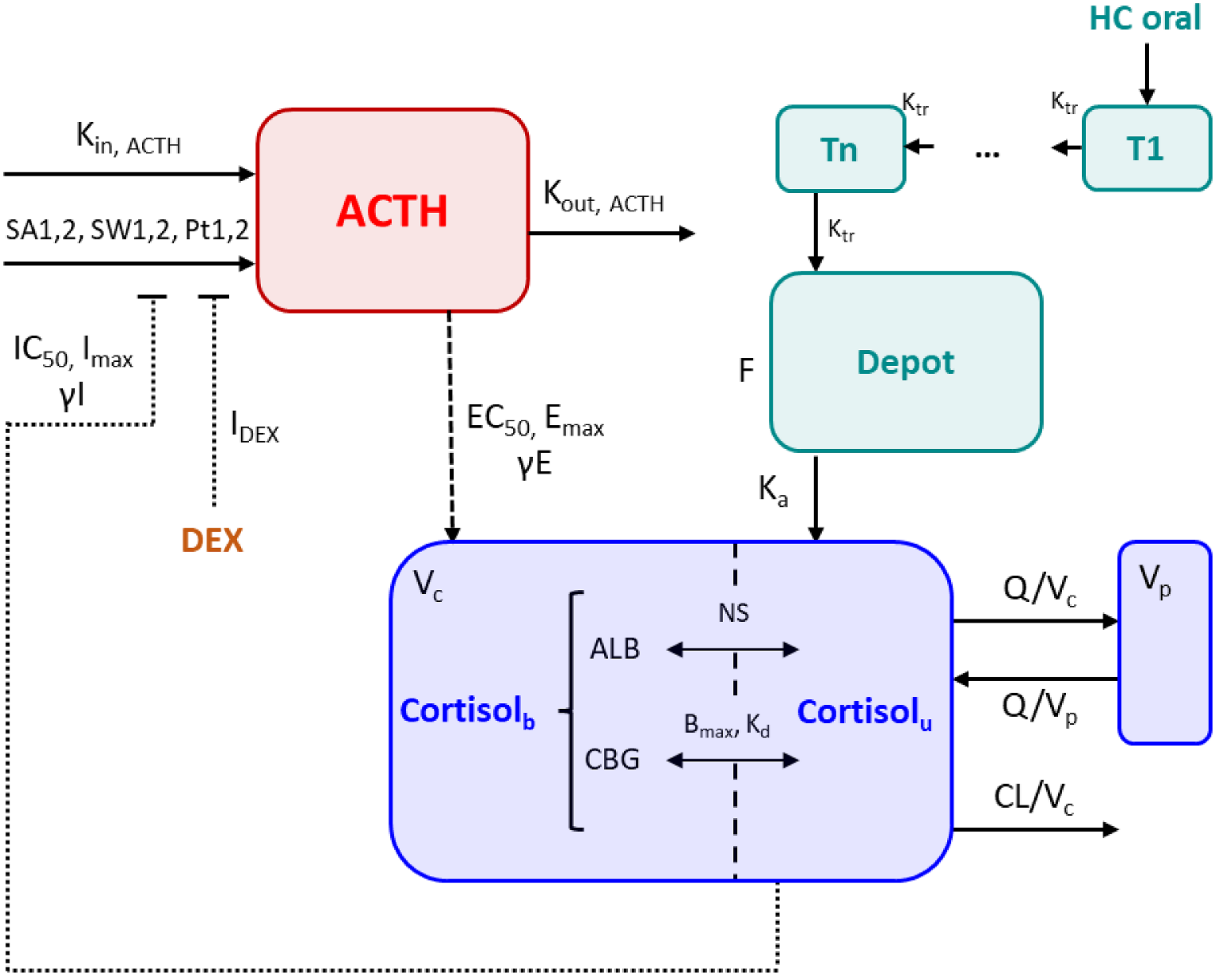
Joint hydrocortisone (cyan part) and endogenous ACTH (red part) and cortisol (blue part) model structure. K_in, ACTH_ = Base*K_out, ACTH_; K_tr_=(N_tr_ + 1)/MTT. ACTH: Adrenocorticotropic hormone, ALB: Albumin, Base: ACTH baseline concentration, B_max_: Maximum binding capacity of CBG, CBG: Corticosteroid binding globulin, CL: Clearance, Cortisol_b_: Bound cortisol, Cortisol_u_: Unbound cortisol, DEX: Dexamethasone, EC_50_: ACTH concentration yielding half-maximum cortisol production, E_max_: Maximum cortisol production rate constant, F: Bioavailability, γE: Hill factor for cortisol production, γI: Hill factor for ACTH suppression, K_a_: Absorption rate constant, K_in, ACTH_: ACTH baseline secretion rate constant, K_d_: Dissociation constant cortisol-CBG, K_out, ACTH_: ACTH elimination rate constant, K_tr_: Transit rate constant, IC_50_: Unbound cortisol concentration yielding half-maximum ACTH suppression, I_DEX_: Dexamethasone-driven ACTH suppression, I_max_: Maximum ACTH suppression by unbound cortisol, MTT: Mean transit time of oral hydrocortisone, N_tr_: Number of transit compartments for oral hydrocortisone absorption, NS: Nonspecific binding cortisol-albumin, Pt1: Peak time morning surge, Pt2: Peak time midday surge, Q: Intercompartmental flow, SA1: Amplitude morning surge, SA2: Amplitude midday surge, SW1: Width morning surge, SW2: Width midday surge, Tn: nth transit compartment, V_c_: Central volume of distribution, V_p_: Peripheral volume of distribution

The ABS1 (“split-dose”) and ABS2 (“estimating number of transit compartments”) models were developed and compared. Both approaches generated conceptually similar models with plausible parameter estimates and both performed better than the original saturable absorption model [20], based on GOF plots (Fig. 3). Yet, the ABS2 model performed better than the ABS1 model (dAIC=-85.8) and was therefore chosen to proceed to Step 2. For the chosen ABS2 model, IIV was included on bioavailability (F), clearance (CL), central and peripheral volume of distribution (V_c_ and V_p_, respectively) and number of transit compartments (N_tr_) (Table 2; Fig. 4, cyan part). Moreover, IOV and a dose-dependency were identified on mean transit time (MTT). Interestingly, both ABS1 and ABS2 models included IIV, IOV and dose-dependency on the common, or relatable, structural PK parameters (Table 2 and S1, Eq. S1-S3).

**Table 2:** Model parameters for ACTH and cortisol (pharmaco)kinetics after each step of the modeling workflow

Parameter [unit]		Parameter estimates			SIR 95%CI
		Hydrocortisone PK model (Step 1, ABS2)	Endogenous model (Step 2)	Final joint model (Step 3)	**Final joint model** **(Step 3)**
*ACTH secretion and elimination*					
SA1 [pmol/h] (70 kg)		-	1160	1300	814–2582
Power BW-SA1^‡^		-	6.27	6.53	4.80–8.28
SW1 [h]		-	0.659	0.606	0.559–0.651
Pt1 [hh: mm]		-	06:24	06:18	06:06–06:24
SA2 [pmol/h]		-	42.1	50.0	31.7–88.4
SW2 [h]		-	2.74	2.33	1.98–2.66
Pt2 [hh: mm]		-	11:36	11:48	11:18 − 12:06
n		-	4*	4*	-
K_out_ [1/h]		-	0.698	0.613	0.434–0.819
Base [pmol/L]		-	1.17	1.29	1.14–1.45
I_DEX_ (%)		-	100*	100*	-
*ACTH-dependent cortisol production*					
EC_50_ [pmol/L]		-	8.85	6.63	5.74–7.94
E_max_ [nmol/h]		-	7880	5400	4466–6605
γE		-	2.61	2.94	2.74–3.17
*Cortisol-dependent ACTH suppression*					
IC_50_ [nmol/L]		-	4.70	4.60	4.22–5.06
I_max_ (%)		-	99.9*	99.9*	-
γI		-	4.62	5.33	4.79–5.95
*Hydrocortisone/Cortisol pharmacokinetics*					
K_a_ [1/h]		13.6	-	24.0	10.4–120
MTT [h] (5 mg dose)		0.787	-	0.868	0.817–0.925
Power Dose-MTT^‡^		0.206	-	0.179	0.133–0.219
N_tr_		2.13	-	2.12	1.76–2.43
F		0.302	0.302*	0.344	0.298–0.379
CL [L/h] (70 kg) ^‡‡^		107	107*	106	98.1–113
V_C_ [L] (70 kg) ^‡‡^		2.03	2.03*	2.15	1.85–2.44
Q [L/h] (70 kg) ^‡‡^		83.2	83.2*	89.9	75.4–104
V_p_ [L] (70 kg) ^‡‡^		54.6	54.6*	61.7	54.9–66.9
NS		4.15*	4.15*	4.15*	-
K_d_ [nmol/L]		9.71*	9.71*	9.71*	-
*Interindividual variability*,* CV (%)*					
ω SA1		-	39.9	44.9	31.0-71.7
ω K_out_		-	52.8	53.7	40.0-87.1
ω Base		-	24.6	24.6	19.1–32.9
ω EC_50_		-	26.5	27.6	22.0-38.4
Covariance Base-EC_50_		-	-	0.0686	0.0384–0.116
ω N_tr_		63.7	-	43.0	31.7–63.8
ω F		52.1	-	48.0	37.4–62.3
ω CL		20.9	-	11.4	8.25–15.7
ω V_c_		18.3	-	-	-
ω V_p_		14.6	-	12.2	9.62–17.3
*Interoccasion variability*,* CV (%)*					
ω MTT		32.4	-	28.6	24.4–33.8
*Residual variability*,* CV (%)*					
σ ACTH_prop_		-	52.9	53.7	51.5–55.2
σ Cortisol_prop_		31.9	51.5	39.5	38.9–40.1

* Fixed parameters

^‡^ Implemented as power covariate model

^‡‡^ Theory-based allometric scaling (exponent = 0.75 for flows and = 1 for volumes)

ACTH: Adrenocorticotropic hormone, ALB: Albumin, Base: ACTH baseline concentration, B_max_: Maximum binding capacity of CBG, CBG: Corticosteroid binding globulin, BW: Body weight, CL: Clearance, Cortisol_b_: Bound cortisol, Cortisol_u_: Unbound cortisol, DEX: Dexamethasone, EC_50_: ACTH concentration yielding half-maximum cortisol production, E_max_: Maximum cortisol production rate constant, F: Bioavailability, γE: Hill factor for cortisol production, γI: Hill factor for ACTH suppression, K_a_: Absorption rate constant, K_in, ACTH_: ACTH baseline secretion rate constant, K_d_: Dissociation constant cortisol-CBG, K_out, ACTH_: ACTH elimination rate constant, K_tr_: Transit rate constant, IC_50_: Unbound cortisol concentration yielding half-maximum ACTH suppression, I_DEX_: Dexamethasone-driven ACTH suppression, I_max_: Maximum ACTH suppression by unbound cortisol, MTT: Mean transit time of oral hydrocortisone, n: Surge functions exponent, N_tr_: Number of transit compartments for oral hydrocortisone absorption, NS: Nonspecific binding cortisol-albumin, Pt1: Peak time morning surge, Pt2: Peak time midday surge, Q: Intercompartmental flow, SA1: Amplitude morning surge, SA2: Amplitude midday surge, SW1: Width morning surge, SW2: Width midday surge, T_n_: nth transit compartment, V_c_: Central volume of distribution, V_p_: Peripheral volume of distribution

### Step 2: endogenous ACTH and cortisol model

ACTH secretion processes and concentration trajectories were best characterized using two surge functions, capturing the morning peak and midday rise, in combination with a baseline secretion: The parameters for a third surge function could not be identified. DEX was assumed to fully suppress ACTH pulsatile secretion (I_DEX_=100%). Interindividual variability was included on the amplitude of the morning surge (SA1), ACTH elimination rate constant (K_out_) and ACTH baseline (Base) (Fig. 4, red part).

Cortisol individual PK parameters were extracted from Step 1 and fixed for each individual (input dataset). The ACTH concentration – cortisol production rate relationship was best characterized by a sigmoidal E_max_ model compared to linear, log-linear and E_max_ effect models (dOFV=-256, -209, -260), respectively. The implementation of the negative feedback loop from unbound cortisol concentrations onto ACTH pulsatile secretion significantly improved the model in terms of OFV: A sigmoidal I_max_ model was chosen as it outperformed the hyperbolic I_max_ model (dOFV=-126). Lastly, IIV was included on EC_50_, the ACTH concentration yielding half-maximum cortisol production.

Following SCM, body weight was found to have an impact on SA1 (amplitude of the morning surge) and no other covariates were retained in the model following backward deletion.

### Step 3: joint model and parameters re-estimation

Merging the developed endogenous ACTH and cortisol models from Step 2 and the refined hydrocortisone PK model from Step 1 (Fig. 4), revealed that none of the parameter estimates and precision changed largely following parameter re-estimation (Table 2). However, IIV on V_c_ was removed from the model as it collapsed to 0 following parameter re-estimation.

For the absorption process of oral hydrocortisone, a power relationship was identified between MTT and dose (exponent = 0.179), resulting in an MTT = 0.57 h for the lowest 0.5 mg dose and MTT = 1.11 h for the highest 20 mg dose. The increased absorption delay at higher doses can be explained by the low aqueous solubility of hydrocortisone (0.35–0.40 mg/mL) [29]. Interindividual variability on F, CL, V_p_ and N_tr_ was low to moderate, CV ≤ 48.0%, as well as IOV on MTT, CV = 28.6% (Table 2).

**Fig. 5:**
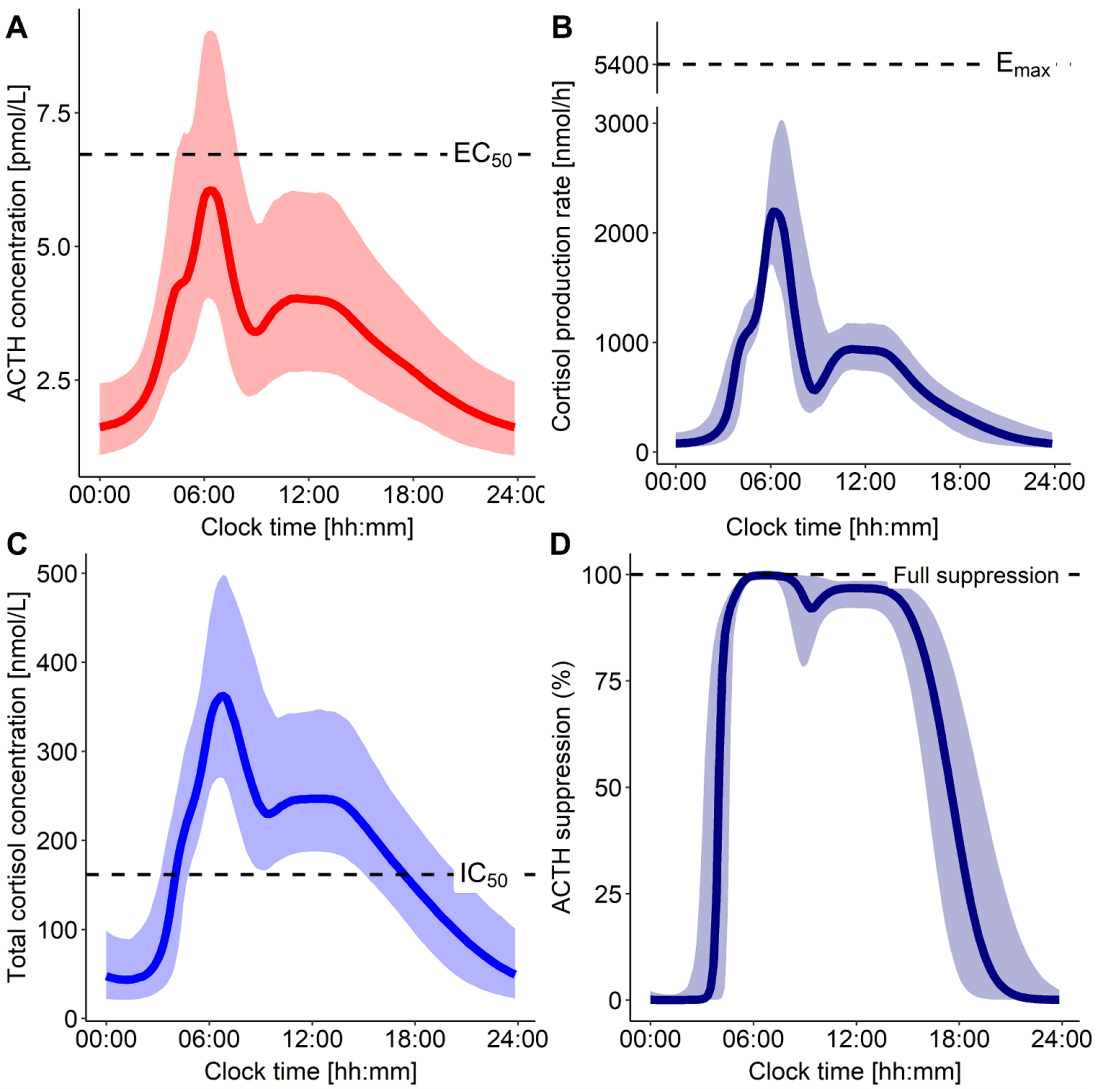
Simulations of 70 kg healthy individuals (*n* = 1000) (**A**) ACTH concentration-time profile and (**B**) subsequent cortisol production rate (y-axis break). (**C**) Total cortisol concentration-time profile and (**D**) subsequent suppression of ACTH pulsatile secretion. Solid lines: Typical profiles. Shaded areas: 90% confidence interval. ACTH: Adrenocorticotropic hormone, EC_50_: ACTH concentration yielding half-maximum cortisol production, E_max_: Maximum cortisol production rate constant, IC_50_: Unbound cortisol concentration yielding half-maximum ACTH suppression

For the ACTH-dependent cortisol production rate, a maximal rate (E_max_) of 5440 nmol/h was estimated, while ACTH concentration yielding half-maximal cortisol production rate (EC_50_) was 6.63 pmol/L (Fig. 5A): Based on these findings, the typical healthy individual (70 kg) did not reach maximal cortisol production rate but remained below 50% of the maximal production rate, i.e. ~2200 nmol/h (Fig. 5B). Estimated baseline ACTH concentrations in this context represented the minimum ACTH concentrations reached at night (Base = 1.17 pmol/L). Possible full suppression of ACTH pulsatile secretion by cortisol was assumed (maximum inhibition (I_max_) fixed to 99.9%), while the unbound cortisol concentration yielding half-maximal ACTH suppression was estimated to 4.60 nmol/L (~ 160 nmol/L total cortisol): The estimated values of IC_50_ = 160 nmol/L and γI = 5.33, implied that the pulsatile secretion of ACTH was almost fully suppressed throughout the entire daytime (~ 05:00–19:00) (Fig. 5C-D) as cortisol concentrations were higher than IC_50_ during the day. Interindividual variability on SA1, K_out_, EC_50_ and Base was moderate to high, CV ≤ 53.6%. Additionally, a high correlation between ACTH baseline and EC_50_ was found and implemented as an off-diagonal element in the IIV matrix (estimated correlation = 95.0%) (Table 2). The power relationship identified between body weight and SA1 explained 58.6% of the IIV on SA1: The estimated exponent was 6.53, resulting in SA1 = 1300 pmol/h for a 70 kg individual and SA1 = 6708 pmol/h for a 90 kg individual. However, as in healthy individuals the pulsatile secretion of ACTH was suppressed before reaching the secretion rate peak, this large difference in ACTH secretion rate peak only translated into smaller differences in ACTH peak concentrations (Fig. S1).

The parameters precision of the joint model evaluated by SIR was considered adequate (RSE ≤ 44.6%) except for K_a_ (RSE ≥ 50.0%), for which the upper confidence interval limit was large (Table 2). However, given that K_a_ was fast (K_a_=24 h^− 1^), larger values would not impact the model predictions and therefore considered acceptable (Fig. S2). GOF plots and VPCs showed no model misspecifications and accurate predictive performance (Fig. S3 and S4 respectively).

### Simulations: CAH patients and healthy individuals

**Fig. 6:**
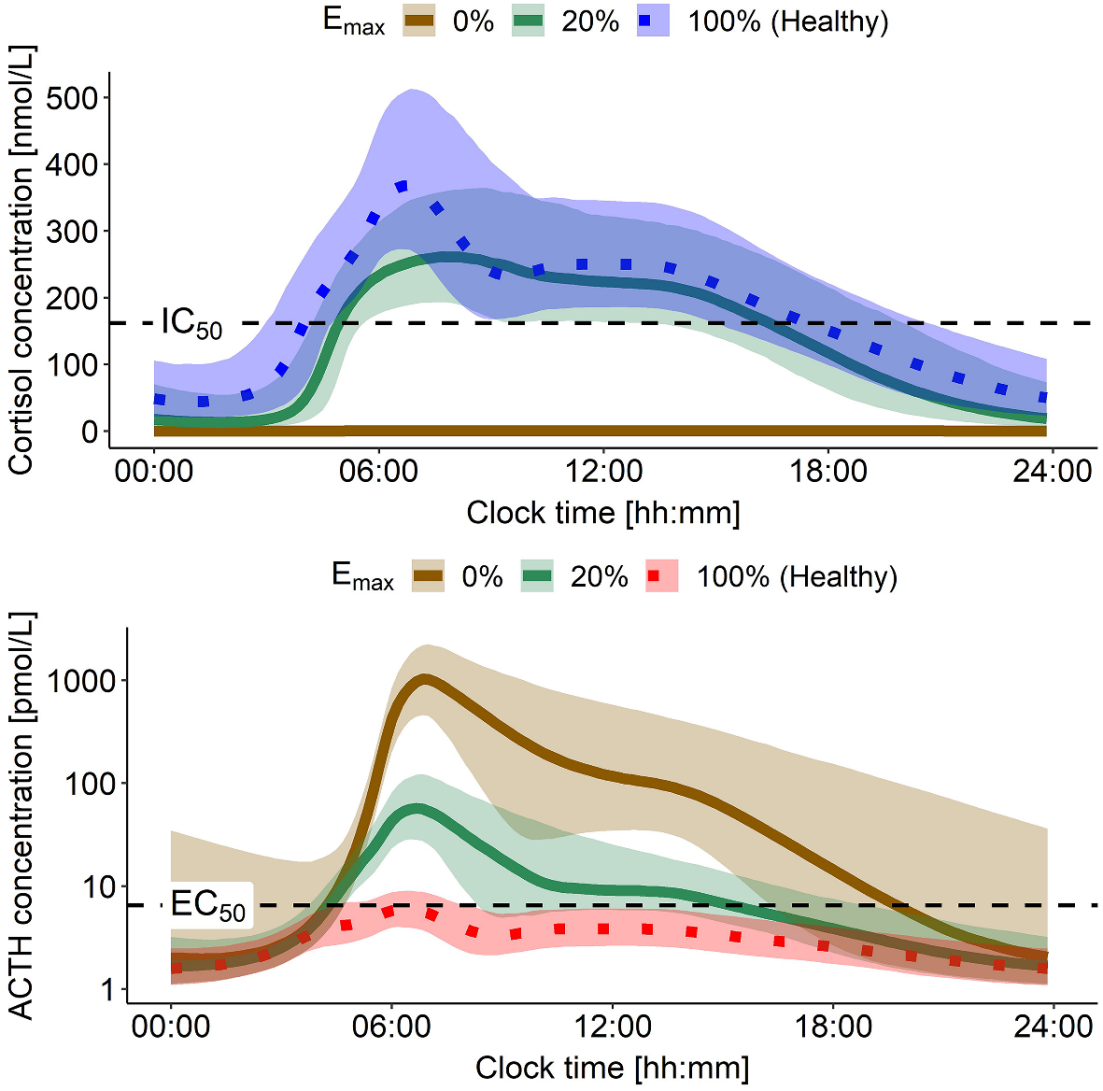
Simulations of 70 kg healthy individuals and CAH patients with mild and severe disease (expressed as different enzymatic activity (E_max_)) (*n* = 1000). Top panel: Cortisol concentrations comparison between healthy state and in CAH patients. Bottom panel: Resulting ACTH concentration time profiles under those conditions; y-axis on log-scale. ACTH: Adrenocorticotropic hormone, EC_50_: ACTH concentration yielding half-maximum cortisol production, E_max_: Maximum cortisol production rate constant, IC_50_: Total cortisol concentration yielding half-maximum ACTH suppression

Simulated patients with mild CAH showed cortisol concentration trajectories throughout the day comparable to the ones observed in simulated healthy individuals (Fig. 6, top). Yet, ACTH morning peak concentrations were around 10-fold higher compared to simulated healthy individuals (Fig. 6, bottom). By contrast, in patients with severe CAH, total cortisol concentrations were negligible as expected, and ACTH morning peak concentrations were around 100-fold higher compared to simulated healthy individuals (Fig. 6, bottom).

### Simulations: interaction with hydrocortisone therapy

**Fig. 7:**
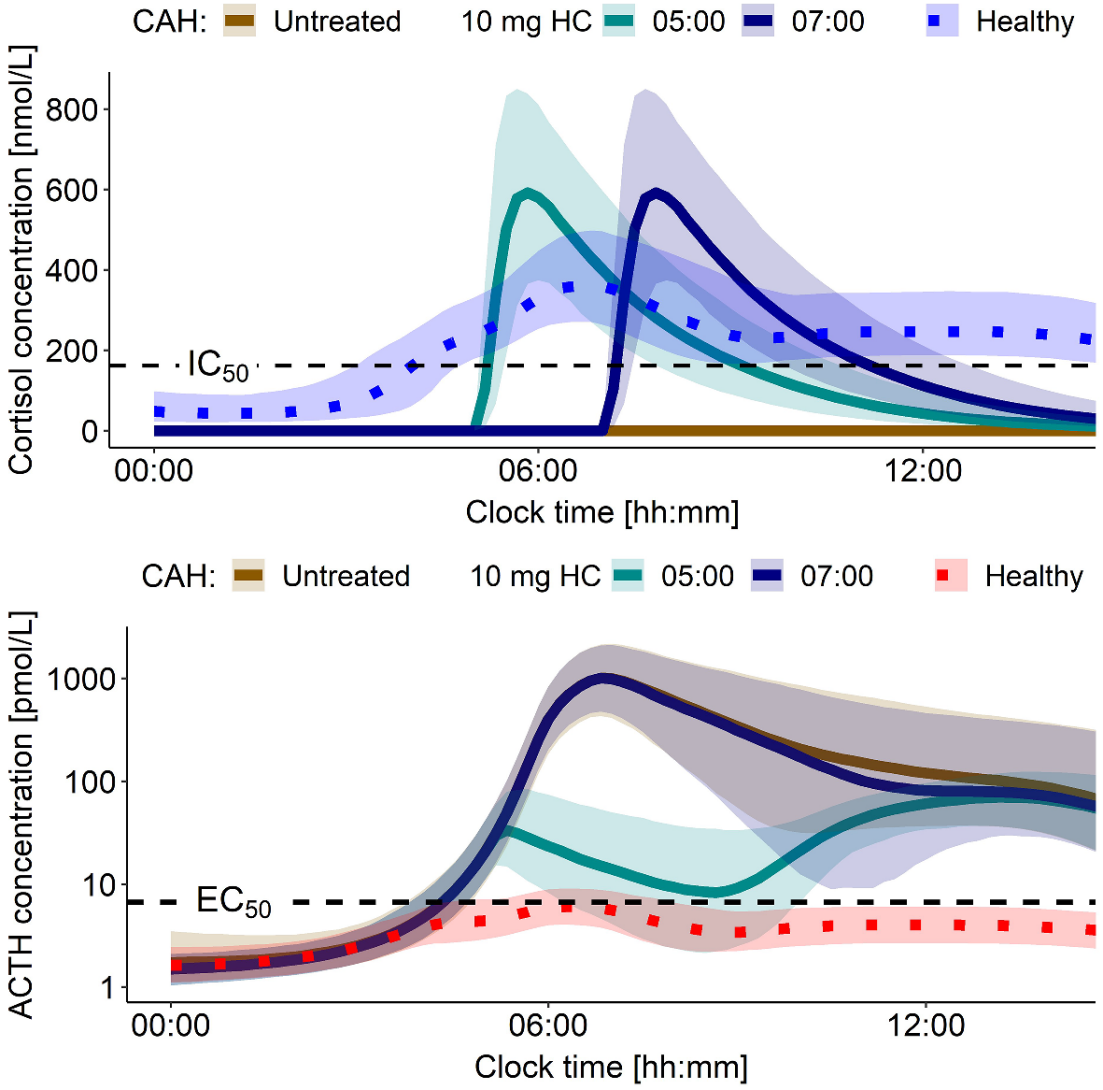
Simulations of 70 kg healthy individuals and severe CAH patients (E_max_=0%) (*n* = 1000). Top panel: Cortisol concentrations comparison between healthy state and in CAH with and without HC administration. Bottom panel: Resulting ACTH concentration time profiles under those conditions; y-axis on log-scale. ACTH: Adrenocorticotropic hormone, CAH: Congenital adrenal hyperplasia, EC_50_: ACTH concentration yielding half-maximum cortisol production, E_max_: Maximum cortisol production rate constant, HC: Hydrocortisone, IC_50_: Total cortisol concentration yielding half-maximum ACTH suppression

To compare ACTH overproduction in adult patients with severe CAH, untreated or with hydrocortisone immediate-release granules administration (10 mg at 05:00 or 07:00), and ACTH secretion in healthy individuals, the developed model was leveraged performing stochastic simulations (*n* = 1000). In patients with severe CAH, no cortisol production was present (Fig. 7, top), therefore no feedback inhibition on ACTH pulsatile secretion was present until and if hydrocortisone was administered (Fig. 7, bottom). Simulated untreated severe CAH patients were found to have around 100-fold higher ACTH morning peak concentrations compared to simulated healthy individuals (Fig. 7, bottom). Similarly, when hydrocortisone was administered too late (i.e. after the ACTH secretion peak time of healthy individuals e.g., at 07:00), ACTH concentration trajectories were similar to the ones in untreated CAH patients (Fig. 7, bottom). However, when hydrocortisone was administered earlier before ACTH peak time at 05:00, the ACTH concentration trajectories were more similar to those in healthy individuals (Fig. 7, bottom). Thus, highlighting the need for dosing hydrocortisone in a timely manner to achieve cortisol concentrations higher than IC_50_ earlier than ACTH peak time secretion to avoid ACTH overproduction: The dosing time of immediate-release hydrocortisone formulations (i.e., not too late) is key in regulating ACTH secretion and must be considered when designing dosing regimens.

## Discussion

In this work, ACTH and cortisol trajectories of CAH patients with different remaining enzymatic activity were successfully generated and the importance of hydrocortisone dosing time to improve cortisol replacement therapy in CAH patients was shown by leveraging the developed quantitative framework that integrated physiological processes and interaction of the HPA axis and hydrocortisone PK.

The obtained hydrocortisone PK modeling results showed delayed absorption of hydrocortisone when higher doses were given, in particular the MTT was 2-fold higher with 20 mg dose compared to 0.5 mg dose. The identified dose-dependent delay in absorption is most likely attributable to hydrocortisone low aqueous solubility [29]. Regarding endogenous ACTH and cortisol modelling results, it appeared that 70 kg healthy individuals did not reach maximal cortisol production rate: The quantification of ACTH-dependent cortisol production could help refining and evaluating the results of ACTH stimulation test, a frequently used test to diagnose adrenal insufficiency [30]. Following cortisol production, the subsequent feedback inhibition from cortisol on ACTH pulsatile secretion was quantified: ACTH pulsatile secretion was found to be mostly fully suppressed during daytime, highlighting the fast action and fine regulation of the feedback inhibition mechanisms of the HPA axis. Moreover, in individuals with larger body weight ACTH peak secretion rate was found to be higher, in fact a strong covariate relationship was identified between SA1 and body weight (power relationship, exponent = 6.53): While this finding was not supported by the literature, we hypothesize it might be a surrogate for differences in ACTH volume of distribution (Fig. S1).

The simulation results showed the capability of the model to simulate CAH patients with different degrees of disease severity. In fact, the results obtained provided a qualitative, yet with quantitative characteristics, agreement with reported clinical phenotype of CAH patients: Patients with mild CAH have been often reported to be asymptomatic (cortisol concentrations following similar trajectories throughout the day) or with signs of androgen excess (increased ACTH, around 10-fold higher morning peak), while for patients with severe CAH excess androgen production was even more pronounced (increased ACTH, around 100-fold higher morning peak) [4, 6, 31, 32]. Additionally, the importance of hydrocortisone dosing time in cortisol replacement therapy was shown: The goal of replacement therapy is to mimic endogenous cortisol circadian rhythm; however, it is essential to consider the impact of therapy on the whole HPA axis dynamics and activity. In fact, from the simulations performed in patients with severe CAH when 10 mg hydrocortisone were given at 05:00 or 07:00, in both cases cortisol concentrations approximated reasonably morning peak cortisol concentrations observed in healthy individuals. Yet, in the studied context only the 05:00 dose achieved cortisol concentrations higher than IC_50_ before ACTH secretion rate peak, and thus reduced ACTH overproduction compared to what was observed in untreated severe CAH patients. This insight highlights the need for dosing hydrocortisone prior to ACTH secretion rate peak time to prevent ACTH, and consequently androgens, overproduction. In this context, the use of modified-release formulations of hydrocortisone should aid the control of the HPA-cortisol pathway in CAH as they better mimic the overnight rise in cortisol compared to immediate-release formulations [33].

This work focused on characterizing the circadian pattern of the HPA axis and cortisol production pathway. However, an ultradian rhythm has been identified, characterized by a more rapid pulsatile glucocorticoid secretion occurring on top of the circadian rhythm [34–37]. A theoretical mathematical model was proposed by Walker et al. in 2010 in which they showed the ultradian behavior could arise by using feed-forward and feedback mechanisms, independently of a supra-pituitary clock [38]. Yet, integrating such behavior in our modeling framework would require much denser sampling than hourly to estimate parameters. In future, a combination of NLME and fully mechanistic modeling approaches might allow to characterize the interplay between circadian and ultradian rhythm. Furthermore, an NLME model for CAH pediatric patients, that included 17-hydroxyprogesterone and androstenedione besides cortisol, was developed by Al-Kofahi et al. in 2021 [39]. However, due to the lack of ACTH measurements, a dual-cosine function was used to characterize circadian fluctuations resulting in reduced physiologically interpretable parameters compared to using surge functions. Similarly, an NLME model for CAH pediatric patients that also included 17-hydroxyprogesterone was developed by Melin et al. in 2020 [40]: Circadian fluctuations in 17- hydroxyprogesterone concentrations were characterized using the sum of two cosine functions. Surge functions were also utilized by Lönnebo et al. in 2007 to characterize the circadian rhythm of ACTH [41]. Yet, the goal of the study was to characterize ACTH suppression by budesonide and identifying cortisol-related ACTH suppression was not possible. Lastly, a mechanistic disposition model for cortisol that included distribution of cortisol in the extravascular compartment was presented by Dorin et al. in 2022 [42]: This process is comparably accounted for in our framework by the presence of a peripheral compartment for cortisol. Additionally, the reported elimination rate constant for unbound cortisol (53.4 h^− 1^) is well in accordance with our estimated value (49.3 h^− 1^).

Some key assumptions were made during model development. Firstly, cortisol production was assumed to be solely dependent on ACTH concentrations, while it is known it could also be produced from cortisone [43]: This could have led to slightly underestimated CL or overestimated cortisol production parameters (EC_50_, E_max_). In addition, it was assumed that unbound cortisol had the potential to fully suppress ACTH pulsatile secretion by fixing I_max_ to 99.9%. While the estimation of I_max_ led to unstable or unidentifiable models, this implied that the feedback inhibition was solely dependent on, or dominated by, unbound cortisol concentrations. This assumption overlooked the known contributions of other glucocorticoids to the feedback mechanism [44, 45]. Nonetheless, given that the concentrations of other glucocorticoids were expected to correlate with cortisol concentrations, using solely cortisol was considered a reasonable approximation. Moreover, during the simulations step with hydrocortisone administration, CAH patients were assumed to not produce any cortisol (E_max_=0), although CAH patients can produce low levels of cortisol depending on the severity of the gene mutation. As the framework was shown to have the potential to simulate patients with different degrees of CAH severity, different combinations of patients and dosing regimens shall be further evaluated in detail. Lastly, we emphasize that only data from a small population (*N* = 13) of healthy participants were used to develop the endogenous ACTH and cortisol submodel. Thus, parameters such as IIV on ACTH secretion peak time could not be estimated despite their physiological plausibility.

The framework has the potential to be further extended: Firstly, the model could be scaled to pediatric patients as they represent the main target population for cortisol replacement therapy with hydrocortisone [14, 18, 20, 46]. However, a quantification of ACTH secretion and consequent cortisol production in children will be needed to develop a pediatric model. Then, biomarkers from the pathway should be included in the model [47] to improve therapy monitoring and the evaluation of the effect of cortisol replacement therapy on the whole pathway respectively.

The simulation step served as proof of concept to demonstrate how the developed framework can be applied to evaluate patients with different degrees of disease severity and to optimize hydrocortisone dosing regimens in cortisol replacement therapy. In the future, simulated ACTH and cortisol concentrations in virtual CAH patients will be validated using real-world patient data when such will become available. The performed simulations will require further work and exploration of different scenarios to produce new dosing regimens recommendation for different patients.

## Conclusions

A quantitative modeling framework was developed integrating and characterizing physiological mechanisms of the HPA axis and cortisol production pathway, as well as hydrocortisone PK. The framework allowed to evaluate the impact of CAH on the HPA axis regulation, plus the additional impact of hydrocortisone administration. The presented model applications, together with the inclusion of further hydrocortisone formulations will allow significant steps forward in CAH therapy individualization and optimization.

## Electronic supplementary material

Below is the link to the electronic supplementary material.

Supplementary Material 1

## Data Availability

The datasets analysed in this study can be shared upon reasonable request.
