# Supplementary material for: A quantitative modeling framework to understand the physiology of the hypothalamic-pituitary-adrenal axis and interaction with cortisol replacement therapy
Source: J Pharmacokinet Pharmacodyn. 2024 Jul 8;51(6):809–24. doi: 10.1007/s10928-024-09934-7 (PMC11579075; doi:10.1007/s10928-024-09934-7)
Supplement: Supplementary file 1 — Supplementary Material 1 [file 10928_2024_9934_MOESM1_ESM.docx]

**Tables:**

Table S1. Parameter estimates for unbound cortisol in ABS1 (“split-dose”) model

| **Parameter [unit]** | **Estimates** | **Parameter description** |
| --- | --- | --- |
| **K_a_ [1/h]** | 2.79 | Absorption rate constant |
| **FA_max_** | 0.932 | Maximum dose fraction absorbed via first-order process |
| **FA_50_ [nmol]** | 11100 | Hydrocortisone amount yielding half-maximum dose fraction absorbed via first-order process |
| **γFA** | -3.68 | Hill factor for dose fraction absorbed via first-order process |
| **K_tr_ [1/h]** | 2.77 | Transit rate constant |
| **F** | 0.285 | Bioavailability |
| **CL [L/h] (70 kg) ^‡^** | 103 | Clearance |
| **V_C_ [L] (70 kg) ^‡^** | 1.76 | Volume of distribution central compartment |
| **Q [L/h] (70 kg) ^‡^** | 61.5 | Intercompartmental flow |
| **V_p_ [L] (70 kg) ^‡^** | 47.6 | Volume of distribution peripheral compartment |
| **NS** | 4.15* | Nonspecific binding cortisol-albumin |
| **K_d_ [nmol/L]** | 9.71* | Dissociation constant cortisol-CBG |
| ***Interindividual variability, CV (%)*** | |  |
| **ω K_tr_** | 25.0 |  |
| **ω F** | 42.8 |  |
| **ω CL** | 22.3 |  |
| **ω V_p_** | 18.0 |  |
| ***Interoccasion variability, CV (%)*** | |  |
| **ω FA** | 6124 |  |
| ***Residual variability, CV (%)*** | |  |
| **σ Cortisol_prop_** | 32.7 |  |

* Fixed parameters

^‡^ Theory-based allometric scaling (exponent=0.75 for flows and =1 for volumes)

**Equations:**

Main equations used in ABS1 (“split-dose”) model:

Eq. S1 FA dose-dependency

$$FA={FA}_{max}*\frac{{Dose}^{\gamma FA}}{{FA}_{50}^{\gamma FA}+{Dose}^{\gamma FA}}$$

Eq. S2 Bioavailability in first depot (first-order absorption)

$$F\left( Depot1 \right)=F*FA$$

Eq. S3 Bioavailability in second depot (transit compartments absorption)

$$F\left( Depot2 \right)=F*(1-FA)$$

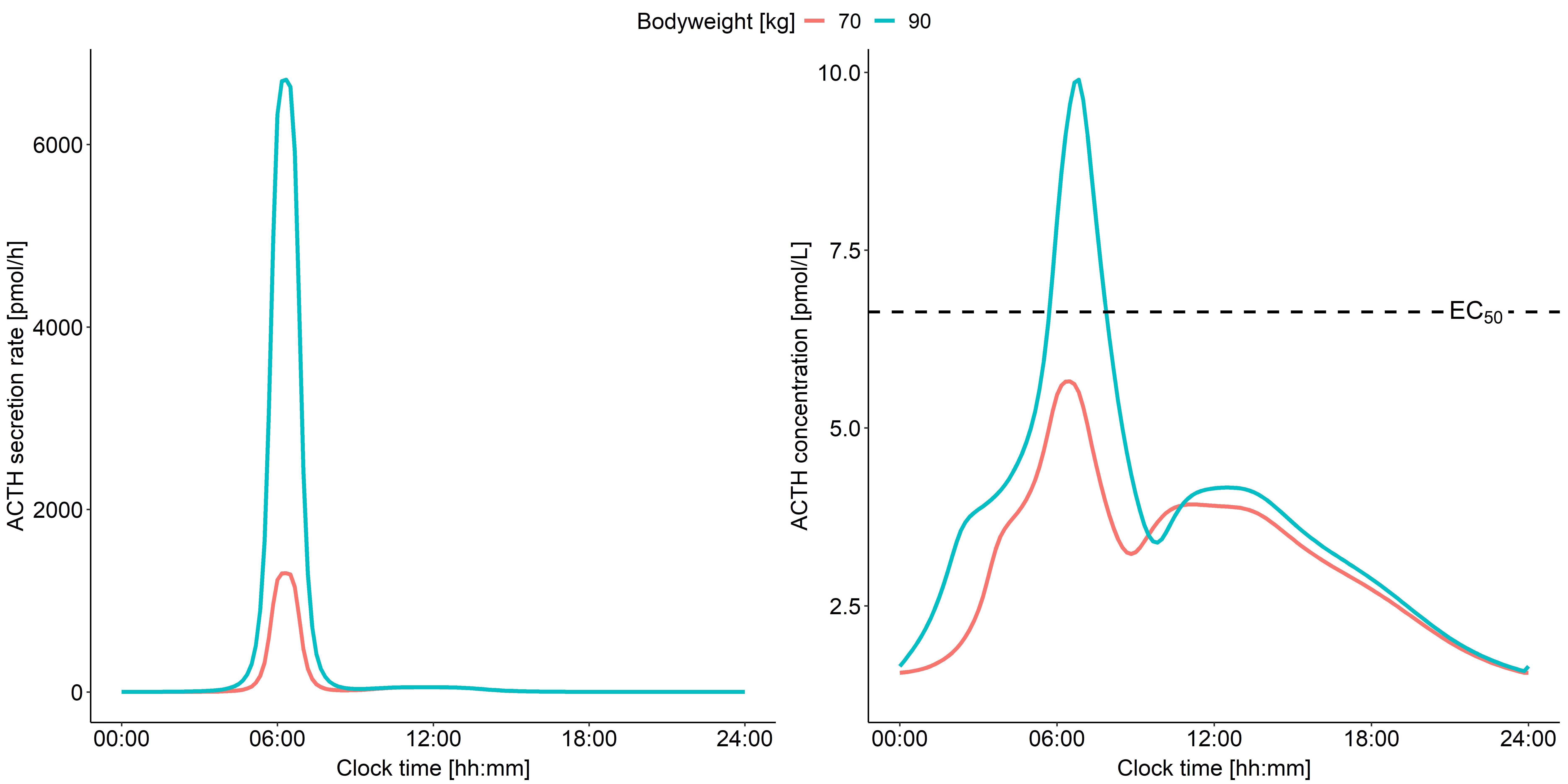
**Figures:**

**Fig. S1** Comparison of differences in ACTH secretion rate (left) and resulting ACTH concentrations (right) in the healthy state for typical 70 kg (red line) and 90 kg (cyan line) individuals.

ACTH: Adrenocorticotropic hormone, EC_50_: ACTH concentration yielding half-maximum cortisol production


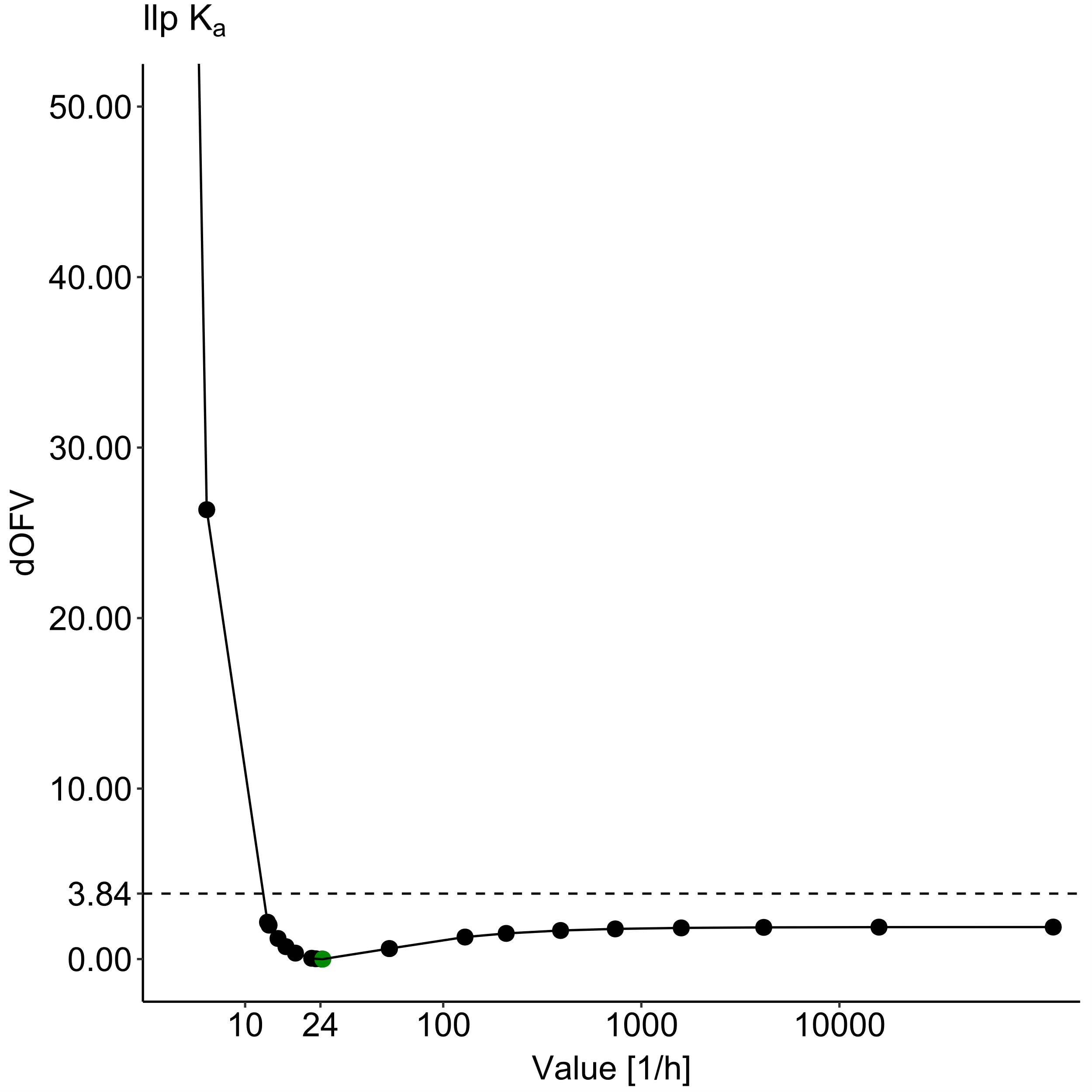


**Fig. S2** Log-likelihood profiling for absorption rate constant (K_a_). Black circles: dOFV resulting from different K_a_ values. Green circle: Original K_a_ value. Dashed line: Threshold for significant difference with one degree of freedom difference and significance level=0.05.

dOFV: Difference in objective function value


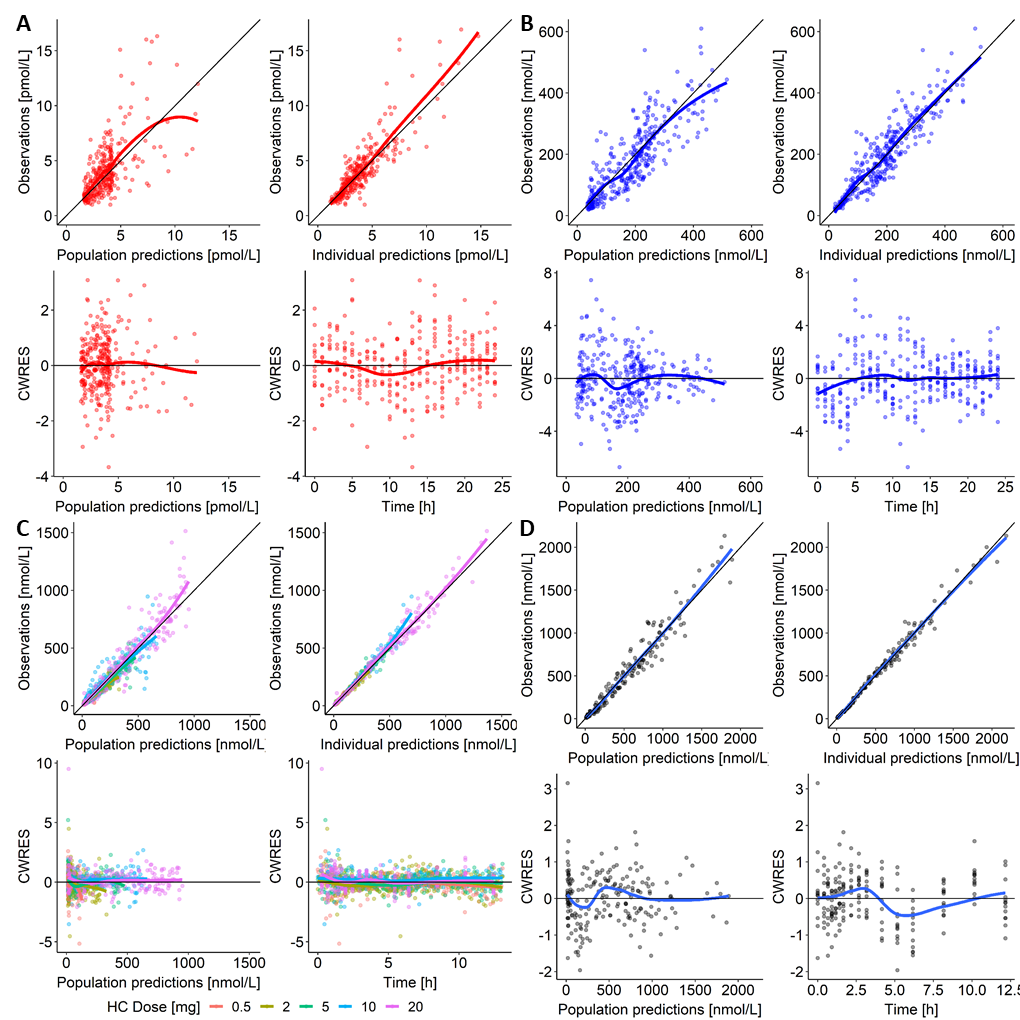


**Fig. S3** Goodness of fit plots for (A) ACTH concentrations, (B) Total cortisol concentrations, (C) Total cortisol concentrations following oral hydrocortisone administration (red: 0.5 mg, yellow: 2 mg, green: 5 mg, blue: 10 mg, violet: 20 mg) and (D) Total cortisol concentrations following intravenous bolus hydrocortisone administration.

CRWES: Conditional weighted residuals, HC: Hydrocortisone


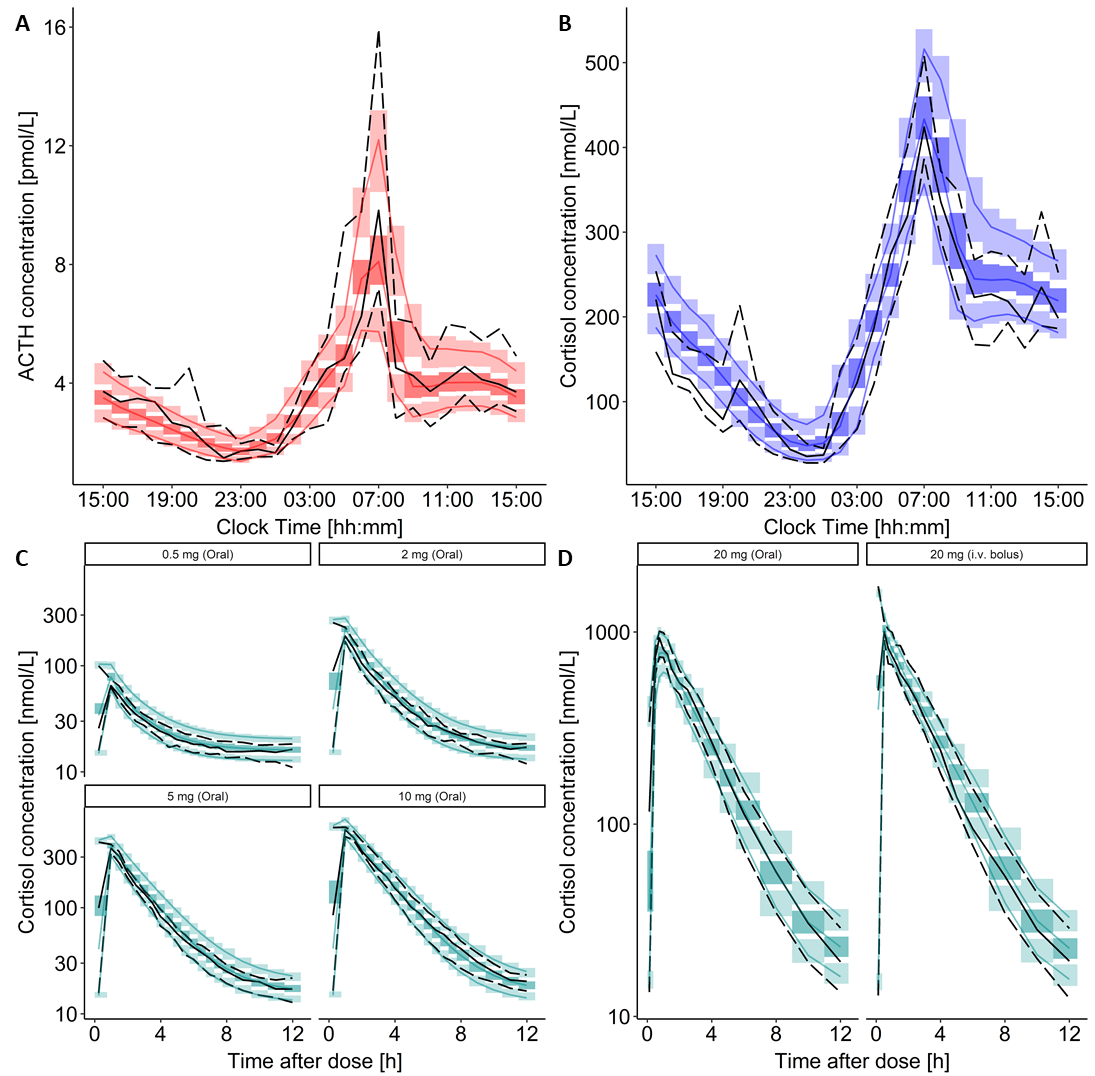


**Fig. S4** Visual predictive check (n=1000) for (A) ACTH concentrations, (B) Total endogenous cortisol concentrations, (C) Total cortisol concentrations following oral hydrocortisone administration in trial 1 and (D) Total cortisol concentrations following hydrocortisone administration in trial 2. Black solid lines: Median observations. Black dashed lines: 5^th^ and 95^th^ percentiles of observations. Colored lines: Median, 5^th^ and 95^th^ percentiles of simulations. Colored boxes: 90% confidence interval around simulated percentiles.

**Model code:**

<https://github.com/Kloft-Lab/Bindellini-et-al._EndogenousACTH-cortisol-HC_PK_NONMEM_script>
